# Hospitalizations for Respiratory Syncytial Virus Among Adults in the United States, 1997–2012

**DOI:** 10.1093/ofid/ofw270

**Published:** 2017-01-09

**Authors:** Susan T. Pastula, Judith Hackett, Jenna Coalson, Xiaohui Jiang, Tonya Villafana, Christopher Ambrose, Jon Fryzek

**Affiliations:** 1 Epidstat Institute, Ann Arbor, Michigan; 2 AstraZeneca/Medimmune, Gaithersburg, Maryland

**Keywords:** adult, hospitalizations, respiratory syncytial virus

## Abstract

**Background:**

Respiratory syncytial virus (RSV) is an established cause of serious lower respiratory disease in children, but the burden in adults is less well studied.

**Methods:**

We conducted a retrospective study of hospitalizations among adults ≥20 years from the 1997–2012 National Inpatient Sample. Trends in RSV admissions were described relative to unspecified viral pneumonia admissions. Hospitalization severity indicators were compared among immunocompromised RSV, non-immunocompromised RSV, and influenza admissions.

**Results:**

An estimated 28237 adult RSV hospitalizations occurred, compared with 652818 influenza hospitalizations; 34% were immunocompromised individuals. Respiratory syncytial virus and influenza patients had similar age, gender, and race distributions, but RSV was more often diagnosed in urban teaching hospitals (73.0% for RSV vs 34.6% for influenza) and large hospitals (71.9% vs 56.4%). Respiratory syncytial virus hospitalization rates increased from 1997 to 2012, particularly for those ≥60, increasing from 0.5 to 4.6 per 100000, whereas unspecified pneumonia admission rates decreased significantly (*P* < .001). Immunocompromised patients with RSV hospitalization had significantly higher inpatient mortality (*P* = .013), use of mechanical ventilation (*P* = .016), mean length of stay (LOS) (*P* < .001), and mean cost (*P* < .001) than non-immunocompromised RSV hospitalizations. Overall, RSV hospitalizations were more severe than influenza hospitalizations (6.2% mortality for RSV vs 3.0% for influenza, 16.7% vs 7.2% mechanical ventilation, mean LOS of 6.0 vs 3.6 days, and mean cost of $38828 vs $14519).

**Conclusions:**

Respiratory syncytial virus hospitalizations in adults are increasing, likely due to increasing recognition and diagnosis. The burden of RSV in adults deserves attention. Although there are fewer hospitalizations than influenza, those that are diagnosed are on average more severe.

Respiratory syncytial virus (RSV) is well established as an important cause of serious lower respiratory disease in children, but the burden in adults is less well studied. Outbreaks identified in long-term care facilities in the late 1970s and early 1980s first drew attention to RSV as a potential cause of serious respiratory disease in older adults [1]. Studies of adults have reported the presence of RSV in 3%–12% of patients hospitalized with respiratory symptoms [2–4]. Respiratory syncytial virus has been confirmed in 1% to 8% of chronic obstructive pulmonary disease (COPD) exacerbations [5–10] and in 5% to 11% of adults admitted with pneumonia, COPD, heart failure, or asthma [2]. More recent evidence suggests that the burden of RSV-related illness among adults may approach or even exceed that of nonpandemic influenza A [2, 11–16].

Older adults are at higher risk for severe morbidity due to RSV [1–3, 11, 16, 17]. From 1995 to 2009 in the United Kingdom, Fleming et al [13] found that older adults made up 79% of hospitalizations for RSV and 93% of deaths from RSV. The annual rate of disease caused by RSV in patients over 65 years of age has been estimated to be between 3% and 7% [2, 18–20]. In an influenza vaccine study of subjects 65 years and older conducted from 2008 to 2010, throat swabs were tested from subjects with influenza-like illness (ILI). Respiratory syncytial virus was found in 7.4% of nonhospitalized cases and in 12.5% of hospitalizations for ILI [3].

Immunocompromised adults, such as bone marrow transplant (BMT) recipients, are also at particularly high risk for severe RSV illness. A study of RSV in immunocompromised patients reported an RSV-related mortality rate of 36% overall and 50% in BMT patients [21]. In another study, mortality rates for BMT patients with RSV ranged from 30% to 70% [4]. Respiratory syncytial virus was diagnosed in 15% of BMT recipients hospitalized for respiratory illnesses, which progressed to pneumonia in 61% of these patients [22]. These studies suggest that significant morbidity and mortality can occur in immunocompromised patients diagnosed with RSV, but more information is needed to demonstrate the role and severity of RSV in adults for the US healthcare system at large.

Inpatient hospital discharge data can be used (1) to examine the importance of diagnosed RSV as a factor in hospitalizations in adults over time and its association with other reported comorbidities and (2) to contextualize these results with influenza as a more commonly recognized cause of severe outcomes in adults with respiratory disease. Our study characterizes the trends in hospitalizations attributed to RSV infections among adults 20 years or older in the United States from 1997 to 2012 using nationally representative inpatient hospital discharge data. We compared severity indicators, including inpatient deaths, use of mechanical ventilations, length of stay (LOS), and costs, among immunocompromised adults with RSV, non-immunocompromised adults with RSV, and adults hospitalized for influenza.

## METHODS

A retrospective study of RSV hospitalizations among adults (≥20 years) was conducted using discharge data from the Nationwide Inpatient Sample (NIS), Healthcare Cost and Utilization Project (HCUP), and Agency for Healthcare Research and Quality (AHRQ) for the years 1997 to 2012. The NIS is a nationally representative sample of hospital inpatient stays developed by the HCUP and sponsored by the AHRQ. Data are contributed from the HCUP State Inpatient Databases (http://www.hcup-us.ahrq.gov/db/hcupdatapartners.jsp) and include records for more than 7 million all-payer hospital stays each year [23].

Nationwide Inpatient Sample data contain up to 25 diagnostic codes for each hospitalization, based on the *International Classification of Diseases, Ninth Revision, Clinical Modification* ([ICD-9-CM] hereafter, simply ICD-9). During the study years, there were 3 RSV-specific ICD-9 codes: 480.1, pneumonia due to RSV; 466.11, bronchiolitis due to RSV; and 079.6, RSV. Our analysis included all hospitalizations occurring in adults ≥20 years of age with at least 1 of the 3 RSV-specific ICD-9 codes listed in any diagnostic position in their hospitalization record.

Hospitalization rates for 1997–2012 were calculated using the weighted estimate of total number of annual hospitalizations due to RSV relative to the US population estimates from annual US Census data. To investigate rates by age, hospitalized individuals were categorized as 20 to 44 years, 45 to 59 years, or 60 years and older. Hospitalization rates were calculated as numbers of cases per 100000 for each age group by year.

We also analyzed rates of RSV compared with those of pneumonia virus unspecified, defined as ICD-9 480.9. The objective was to understand whether any observed changes in RSV hospitalization incidence were driven by changes in RSV diagnosis frequency or true disease incidence.

For comparison of disease burden, we also assessed adult hospitalizations for influenza, defined as ICD-9 codes 487–488. If a hospitalization had codes for both RSV and influenza, it was counted toward both populations. To examine the burden of disease for RSV and influenza, we compared distribution by hospital characteristics such as geographical area, hospital type (urban teaching/urban nonteaching/rural), and bedsize, using definitions included in the NIS database [23].

We analyzed disease severity using several indicators recorded in the NIS, including inpatient mortality, LOS, use of mechanical ventilation, and total cost. Costs were adjusted to 2015 US dollars. These indicators were examined for the entire study period and by year to examine potential time trends. To investigate any temporal bias effects, we repeated the analyses of hospital characteristics and severity after restricting the data to only the 3 most recent years. Because they were expected to experience differences in disease outcomes, hospitalization records were classified as being in adults who were immunocompromised or not based on an existing AHRQ algorithm based on ICD-9-CM diagnosis and procedure codes [24]. Individuals’ hospitalization records were classified as immunocompromised if they had at least 1 of these qualifying diagnostic or procedural codes in any position.

All data management and statistical analyses for this study were carried out using SAS version 9.3 (SAS Institute Inc., Cary, NC), with procedures that incorporated NIS-provided weights to account for the structure of the sample survey data. There was no imputation of missing values in any analysis. The temporal trend in incidence rates by age group was assessed by Poisson regression. Logistic regression was used to examine temporal trends in mechanical ventilation and inpatient mortality. Because lengths of hospitalization stay and total hospitalization charges were not normally distributed, geometric means were calculated. Linear regression was used to test temporal trends in lengths of hospitalization stay and total hospitalization charges.

## RESULTS

### Patient Characteristics

A total of 28237 RSV inpatient hospitalizations were identified in adults in the 1997 to 2012 NIS database, distributed as follows: 480.1, pneumonia due to RSV (43.6%); 466.11, bronchiolitis due to RSV (11.4%); and 079.6, RSV (47.6%). A total of 33.6% of RSV admissions were classified as immunocompromised (Table 1). Overall, RSV hospitalizations were more common in those who were 60 and older (57.8%), female (56.6%), and non-Hispanic white (60.8%). Non-immunocompromised RSV patients were more often female (62.1%), but immunocompromised RSV patients were less often female (45.7%). A higher proportion (80.7%) of adult RSV hospitalizations occurred in the winter months between December and March than in the other 8 months of the year (data not shown). An association was observed between RSV hospitalization and immunocompromised status.

**Table 1.** Characteristics of RSV Patients, 1997–2012, Compared to Influenza Patients by Immunocompromised Status and Pneumonia Virus Unspecified

Characteristic	RSV n (%) IC	RSV n (%) Not IC	RSV n (%) Total	Influenza n (%) IC	Influenza n (%) Not IC	Influenza n (%) Total	Pneumonia, Virus Unspec. n (%) Total
Total	9483 (33.6%)	18754 (66.4%)	28237 (100%)	66944 (10.3%)	585874 (89.7%)	652818 (100%)	149433 (100%)
Age (years)							
20–44	2157 (22.7%)	2904 (15.5%)	5061 (17.9%)	12705 (19.0%)	125845 (21.5%)	138549 (21.2%)	32758 (21.9%)
45–59	3208 (33.8%)	3653 (19.5%)	6861 (24.3%)	16510 (24.7%)	113842 (19.4%)	130352 (20.0%)	33794 (22.6%)
60+	4118 (43.4%)	12198 (65.0%)	16316 (57.8%)	37729 (56.4%)	346188 (59.1%)	383916 (58.8%)	82882 (55.5%)
Gender							
Male	5147 (54.3%)	7108 (37.9%)	12255 (43.4%)	35634 (53.3%)	232604 (39.7%)	268238 (41.1%)	60280 (40.4%)
Female	4331 (45.7%)	11646 (62.1%)	15977 (56.6%)	31282 (46.7%)	353060 (60.3%)	384342 (58.9%)	89109 (59.6%)
Race or Ethnic Group							
Non-Hispanic White	5744 (60.6%)	11413 (60.9%)	17157 (60.8%)	34309 (51.3%)	339918 (58.0%)	374228 (57.3%)	91406 (61.2%)
Non-Hispanic Black	1209 (12.8%)	1856 (9.9%)	3065 (10.9%)	10447 (15.6%)	48015 (8.2%)	58462 (9.0%)	11871 (7.9%)
Hispanic	735 (7.8%)	1220 (6.5%)	1955 (6.9%)	5623 (8.4%)	35440 (6.0%)	41063 (6.3%)	8016 (5.4%)
Other or Unknown	1794 (18.9%)	4265 (22.7%)	6060 (21.5%)	16564 (24.7%)	162501 (27.7%)	179065 (27.4%)	38140 (25.5%)

Abbreviations: IC, immuncompromised; RSV, respiratory syncytial virus; Unspec., unspecified.

Influenza was more commonly diagnosed in the NIS than was RSV: 652818 influenza patients were identified, nearly 23 times the number of RSV hospitalizations. The vast majority of influenza cases were in patients that were not immunocompromised (89.7%), but the age, gender, and racial distributions of patients with influenza were not appreciably different than those of RSV (Table 1). Six hundred eighty-three hospitalizations had both RSV and influenza diagnosis codes, equaling 2.4% of all RSV admissions and 0.1% of all influenza admissions.

A total of 149433 admissions for pneumonia, virus unspecified were identified between 1997 and 2012. Patients with this diagnosis were more often 60 and older (55.5%), female (59.6%), and non-Hispanic white (61.2%).

### Hospital Characteristics

Both RSV and influenza hospitalizations were slightly more likely to be from the southern United States than other regions of the country and slightly less likely from the West (Table 2). Respiratory syncytial virus cases were identified most often in urban teaching hospitals (73.0%) and large hospitals (71.9%) as opposed to smaller facilities (9.9%). An even larger proportion of immunocompromised RSV patients were diagnosed in urban teaching hospitals (88.8%) and large hospitals (80.5%). In contrast, influenza hospitalizations were more evenly distributed between rural (26.2%), urban nonteaching (39.2%), and urban teaching hospitals (34.6%). Immunocompromised influenza patients were most often seen in urban teaching hospitals (51.1%), but those who were not immunocompromised were commonly treated in urban nonteaching (39.6%) and urban teaching (32.8%) hospitals. Pneumonia virus unspecified patients had similar distributions to influenza; they were most often from the southern United States (37.4%). They were identified in urban nonteaching hospitals most frequently (42.5%) and large hospitals (57.1%).

**Table 2.** Hospital Characteristics of RSV, 1997–2012, Compared to Influenza by Immuncompromised Status and Pneumonia Virus Unspecified

Hospital Characteristic	RSV n (%) IC	RSV n (%) Not IC	RSV n (%) Total	Influenza n (%) IC	Influenza n (%s) Not IC	Influenza n (%) Total	Pneumonia, Virus Unspec. n (%) Total
Total	9483 (33.6%)	18754 (66.4%)	28237 (100%)	66944 (10.3%)	585874 (89.7%)	652818 (100%)	149433 (100%)
Region							
NE	1954 (22.7%)	4527 (26.3%)	6481 (25.1%)	11382 (18.2%)	89130 (15.9%)	100512 (16.1%)	20721 (14.4%)
Midwest	2041 (23.7%)	4841 (28.1%)	6882 (26.6%)	18462 (29.5%)	174603 (31.1%)	193065 (30.9%)	37056 (25.7%)
South	2639 (30.6%)	4610 (26.8%)	7249 (28.1%)	22001 (35.2%)	215460 (38.4%)	237461 (38.1%)	54028 (37.4%)
West	1983 (23.0%)	3247 (18.9%)	5231 (20.2%)	10684 (17.1%)	82267 (14.7%)	92951 (14.9%)	32582 (22.6%)
Location							
Rural	126 (1.5%)	1615 (9.5%)	1741 (6.8%)	8536 (13.7%)	154446 (27.6%)	162982 (26.2%)	33010 (23%)
Urban nonteaching	832 (9.7%)	4336 (25.4%)	5168 (20.2%)	21901 (35.2%)	221756 (39.6%)	243657 (39.2%)	61093 (42.5%)
Urban teaching	7612 (88.8%)	11091 (65.1%)	18703 (73.0%)	31806 (51.1%)	183351 (32.8%)	215157 (34.6%)	49672 (34.5%)
Bedsize							
Small	503 (5.9%)	2043 (12.0%)	2546 (9.9%)	7400 (11.9%)	105106 (18.8%)	112506 (18.1%)	22419 (15.6%)
Medium	1171 (13.7%)	3477 (20.4%)	4648 (18.1%)	12950 (20.8%)	145675 (26.0%)	158625 (25.5%)	39279 (27.3%)
Large	6896 (80.5%)	11523 (67.6%)	18419 (71.9%)	41892 (67.3%)	308772 (55.2%)	350664 (56.4%)	82076 (57.1%)

Abbreviations: IC, immuncompromised; NE, North East; RSV, respiratory syncytial virus; Unspec., unspecified.

### Trends

**Figure 1.**
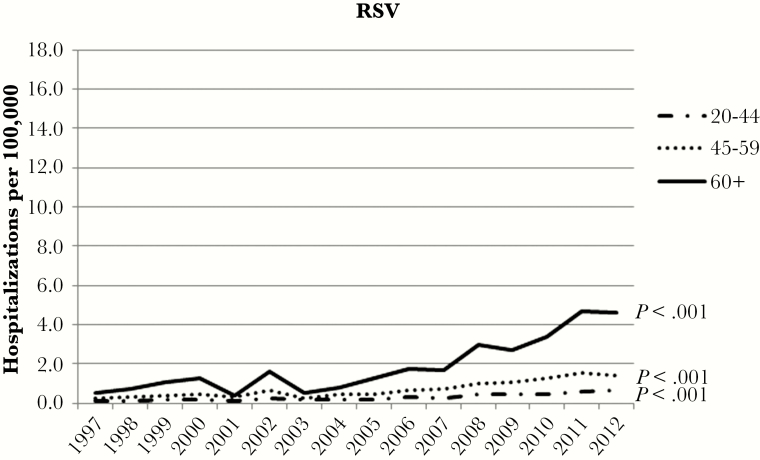
Trends in respiratory syncytial virus (RSV) hospitalization rates by year and age group, 1997–2012.

**Figure 2.**
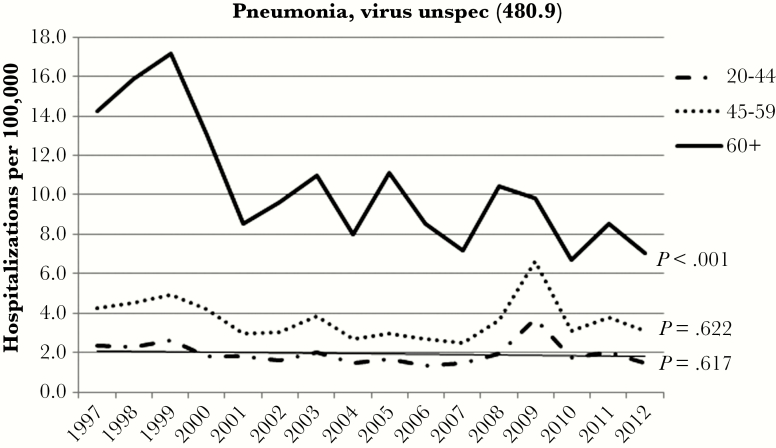
Trends in pneumonia, virus unspecified ([unspec.] *International Classification of Diseases, Ninth Revision, Clinical Modification* 480.9) by year and age group, 1997–2012.

Figure 1 shows the annual age-stratified RSV hospitalization rates per 100000 persons. The rate of RSV hospitalizations increased significantly for all ages between 1997 and 2012 (*P* < .001), most dramatically so for patients 60 years and older. The hospitalization rate in this group increased by 8.6-fold from 0.54 to 4.64 per 100000 over the 16-year period. In contrast to the RSV rates, there was a significant decrease in pneumonia virus unspecified admissions among those 60 and older in the same timeframe (*P* < .001) (Figure 2). Pneumonia virus unspecified admission rates among 20- to 44-year-olds and 45- to 59-year-olds had nonsignificant decreases (*P* = .617 and *P* = .622, respectively).

### Indicators of Severity and Hospitalization Costs

Frequency of death increased with age, from 4.1% of 20- to 44-year-olds with RSV to 6.2% of 45- to 59-year-olds to 6.9% of inpatients 60 and older (Table 3). Thus, the majority of all RSV-related deaths occurred amongst those 60 and older (63.8% = 1118 of 1751 total RSV deaths). However, mechanical ventilation use, LOS, and costs were highest in 45- to 59-year-olds. Compared with non-immunocompromised patients, immunocompromised patients with RSV were more likely to die during hospitalization (7.3% vs 5.6%, *P* = .013), had longer mean LOS (7.3 days vs 5.4 days, *P* < .001), and had higher mean cost ($66476 vs $29316, *P* < .001), but were less likely to receive mechanical ventilation (14.6% vs 17.7%, *P* = .016).

**Table 3.** Indicators of Severity for RSV Hospitalizations, 1997–2012, Compared to Influenza Hospitalizations by Immuncompromised Status and Pneumonia Virus Unspecified

Severity Indicator	RSV n (%) IC	RSV n (%) Not IC	RSV n (%) Total	Influenza n (%) IC	Influenza n (%) Not IC	Influenza n (%) Total	Pneumonia Virus Unspec. n (%) Total
Total Hospitalizations	9483 (33.6%)	18754 (66.4%)	28237 (100%)	66944 (10.3%)	585874 (89.7%)	652818 (100%)	149433 (100%)
Died during hospitalization	695 (7.3%)	1056 (5.6%)	1751 (6.2%)	3833 (5.7%)	15873 (2.7%)	19706 (3.0%)	5359 (3.6%)
Age 20–44	139 (6.4%)	71 (2.4%)	210 (4.1%)	507 (4.0%)	1519 (1.2%)	2026 (1.5%)	514 (1.6%)
Age 45–59	267 (8.3%)	156 (4.3%)	423 (6.2%)	923 (5.6%)	2321 (2.0%)	3244 (2.5%)	706 (2.1%)
Age 60+	289 (7.0%)	828 (6.8%)	1118 (6.9%)	2402 (6.3%)	12033 (3.5%)	14436 (3.8%)	4139 (5.0%)
Mechanical ventilation use	1389 (14.6%)	3319 (17.7%)	4708 (16.7%)	7899 (11.8%)	38877 (6.6%)	46777 (7.2%)	13449 (9.0%)
Age 20–44	262 (12.1%)	452 (15.6%)	714 (14.1%)	1380 (10.9%)	8131 (6.5%)	9510 (6.9%)	3127 (9.5%)
Age 45–59	469 (14.6%)	810 (22.2%)	1279 (18.6%)	2475 (15.0%)	10655 (9.4%)	13130 (10.1%)	3670 (10.9%)
Age 60+	657 (16.0%)	2058 (16.9%)	2715 (16.6%)	4045 (10.7%)	20092 (5.8%)	24137 (6.3%)	6652 (8.0%)
Length of stay (days)^a^	7.3	5.4	6.0	4.8	3.5	3.6	4.3
Age 20–44	7.4	4.6	5.6	4.1	2.6	2.7	3.6
Age 45–59	7.5	5.3	6.2	4.8	3.3	3.5	4.2
Age 60+	7.1	5.6	6.0	5.1	4.0	4.1	4.7
Adjusted cost ($)^a^	66476.1	29316.2	38827.6	25198.5	13634.9	14519.1	18051.2
Age 20–44	71822.3	27021.1	41202.0	24070.5	11831.5	12627.4	16950.0
Age 45–59	73896.5	33196.5	48431.2	29294.9	14761.9	16098.0	19473.9
Age 60+	58648.8	28785.8	34609.1	23958.1	13985.2	14742.5	17940.8

Abbreviations: IC, immuncompromised; RSV, respiratory syncytial virus; Unspec., unspecified.

^a^Geometric mean.

There were more than 10 times as many inhospital deaths with diagnoses of influenza compared with RSV, but death occurred in only 3.0% of influenza hospitalizations and 3.6% of pneumonia virus unspecified hospitalizations compared with 6.2% of RSV hospitalizations. The average LOS for RSV was 6.0 days, compared with 3.6 days for influenza, and 4.3 days for pneumonia virus unspecified. Relatedly, the mean adjusted costs for RSV hospitalizations were higher ($38828) than those for influenza ($14519) or unspecified pneumonia ($18051) hospitalizations.

When more recent data (2010–2012) were examined separately, the patterns of severity were similar to those observed in the full 16-year dataset (Supplemental Table 1). Recent RSV hospitalizations had lower mortality than the full dataset (5.1% vs 6.2%) but higher mechanical ventilation use (17.5% vs 16.7%). Although the average LOS from 2010 to 2012 was slightly shorter than the overall dataset (5.6 days vs 6.0 days), the mean adjusted cost per case was higher for recent RSV at $43328, compared with $38828. The higher severity of RSV versus influenza hospitalizations was also present in 2010–2012, with higher frequency of mortality (5.1% for RSV vs 3.3% for influenza), more frequent use of mechanical ventilation (17.5% vs 11.1%), longer LOS (5.6 vs 3.6 days), and higher cost per hospitalizations ($43328 vs $22631).

## DISCUSSION

In our study of adults hospitalized for RSV, identified by use of ICD-9 codes, we found an abrupt increase in the rate of hospitalizations of RSV starting approximately in 2007, especially in older adults (≥60 years). We observed an overall decrease in rates of pneumonia virus unspecified for this age group, which suggests that this pattern is due to increased recognition (and diagnosis) of RSV as an important cause of disease in older adults rather than true increases in incidence. This is also consistent with our observation that RSV was identified more often in urban teaching hospitals than in smaller or rural hospitals, although this could also (1) be related to transfer of complex cases to tertiary care centers or (2) due to molecular diagnostic testing being more accessible in a teaching facility. As awareness and testing for RSV in adults continues to increase, more effective treatments can be administered, likely limiting improper use of antibiotics.

Although counts of diagnosed RSV hospitalizations in adults are still dwarfed by those of influenza, individuals hospitalized with RSV had more severe illness compared with those hospitalized with influenza. This observation of elevated severity is consistent with data from other sources. A prospective study conducted between 1975 and 1995 in healthy working-age adults found the duration of RSV illness to be longer than that of influenza (9.5 days vs 6.8 days) [19, 25]. In our analysis, 16.7% of RSV hospitalizations required mechanical ventilation, similar to a 2010 review that reported that 3.2%–13% of adult RSV patients required assisted ventilation [26]. Because it is possible that more severe cases are more likely to be diagnosed, further evidence is needed to confirm the degree of severity in RSV hospitalizations observed in this study as diagnosis becomes more common, but the present findings suggest the importance of increased awareness that RSV can be an important cause of disease in adults. These results also provide further evidence that the risk of serious RSV disease is particularly high among immunocompromised adults, consistent with previous observations [4, 21, 22].

Our study methodology had several inherent limitations. Using ICD-9-coded administrative data will underestimate RSV incidence, because these are the most symptomatic cases in the community needing hospitalization. Not all hospitalizations receive diagnostic testing to identify the causal pathogen. The burden of RSV disease is particularly likely to be underestimated in adults, because testing adult respiratory specimens for RSV is generally not standard practice. Several authors have reported that RSV infections are rarely diagnosed in adults, in part because available rapid antigen detection tests were insensitive in adults and because medical practitioners rarely request tests for RSV for this age group [1, 17, 27]. The trend in increasing diagnosis of RSV may have resulted from the development of improved sensitivity in diagnostic testing in recent years, causing the demonstrated shift in cases from another respiratory diagnosis to RSV.

Although lack of diagnostic testing may have led to underestimation of the total number of hospitalizations related to RSV, these codes are the most specific method of identifying RSV-related hospitalizations in data based on ICD-9 codes alone. All diagnostic code positions were used to prevent underestimations and biases related to variability in decisions about diagnostic priority across regions and more than a decade of data.

The strength of this study lies in the size and quality of the database analyzed. The NIS database is a representative sample of all hospitalizations in the United States, so this analysis of the hospital admissions for RSV, influenza, and non-RSV pneumonia is generalizable to the total US population. Fifteen years of data provided the ability to look at trends across years, and this allowed us to analyze healthcare use patterns by geographic area, as well as hospital type and size. Because of the large amount of data in the NIS, examining a relatively rarely reported disease such as RSV is possible.

## CONCLUSIONS

Respiratory syncytial virus is a serious respiratory disease in children that is being increasingly recognized as a serious cause of illness in adults. The incidence of diagnosed RSV hospitalizations, especially in older adults, has increased significantly in the United States. As clinicians become more aware of and diagnose more cases of RSV, rates may continue to climb. Respiratory syncytial virus hospitalizations appear to be consistently more severe than influenza hospitalizations, especially for older adults and those that are immunocompromised, with greater clinical and economic burden. Respiratory syncytial virus disease deserves attention as a potentially severe cause of respiratory hospitalizations in adults.

## Supplementary Data

Supplementary materials are available at *Open Forum Infectious Diseases* online. Consisting of data provided by the authors to benefit the reader, the posted materials are not copyedited and are the sole responsibility of the authors, so questions or comments should be addressed to the corresponding author.

## Supplementary Material

ofw270_suppl_supplementary_tables
