# Supplementary material for: Hospitalizations for Respiratory Syncytial Virus Among Adults in the United States, 1997–2012
Source: Open Forum Infect Dis. 2017 Jan 9;4(1):ofw270. doi: 10.1093/ofid/ofw270 (PMC5414053; doi:10.1093/ofid/ofw270)
Supplement: ofw270_suppl_supplementary_tables [file ofw270_suppl_supplementary_tables.docx]

**Supplementary Tables**

Supplementary Table 1 - Indicators of Severity for RSV Hospitalizations, 2010-2012, compared to Influenza hospitalizations by Immunocompromised (IC) status and Pneumonia virus unspecified

| Severity Indicator | RSV n (%)  IC | RSV n (%)  Not IC | RSV n (%)  Total | Influenza n (%)  IC | Influenza n (%)  Not IC | Influenza n (%)  Total | Pneumonia virus unspecified n (%) Total |
| --- | --- | --- | --- | --- | --- | --- | --- |
| Total | 3,865 | 8,342 | 12,206 | 18,261 | 108,742 | 127,002 | 25,175 |
| Died during hospitalization | 193 (5.0%) | 427 (5.1%) | 620 (5.1%) | 1,119 (6.1%) | 3,102 (2.9%) | 4,221 (3.3%) | 848 (3.4%) |
| Age 20-44 | 30 (4.0%) | 23 (2.0%) | 53 (2.8%) | 139 (4.7%) | 424 (1.8%) | 563 (2.1%) | 96 (1.7%) |
| Age 45-59 | 68 (5.7%) | 46 (2.9%) | 114 (4.1%) | 272 (5.8%) | 611 (2.6%) | 883 (3.1%) | 156 (2.4%) |
| Age 60+ | 95 (4.9%) | 358 (6.3%) | 453 (6.0%) | 708 (6.7%) | 2067 (3.4%) | 2,775 (3.9%) | 595 (4.5%) |
| Mechanical ventilation use | 580 (15.0%) | 1,551 (18.6%) | 2,132 (17.5%) | 2,788 (15.3%) | 11,350 (10.4%) | 14,138 (11.1%) | 3,163 (12.6%) |
| Age 20-44 | 83 (11.2%) | 202 (17.9%) | 285 (15.3%) | 430 (14.5%) | 2,366 (9.8%) | 2,796 (10.3%) | 754 (13.7%) |
| Age 45-59 | 186 (15.6%) | 349 (22.2%) | 535 (19.3%) | 881 (18.8%) | 3,345 (14.3%) | 4,226 (15.1%) | 928 (14.3%) |
| Age 60+ | 311 (16.1%) | 1,000 (17.7%) | 1,311 (17.3%) | 1,477 (13.9%) | 5,639 (9.2%) | 7,116 (9.9%) | 1480 (11.2%) |
| Length of stay (days)^1^ | 6.6 | 5.2 | 5.6 | 4.8 | 3.5 | 3.6 | 4.1 |
| Age 20-44 | 6.3 | 4.8 | 5.4 | 4.3 | 2.8 | 2.9 | 3.6 |
| Age 45-59 | 6.6 | 5.0 | 5.6 | 4.9 | 3.6 | 3.8 | 4.1 |
| Age 60+ | 6.8 | 5.3 | 5.7 | 4.8 | 3.8 | 3.9 | 4.3 |
| Adjusted cost ($)^1^ | 66,336.2 | 35,175.6 | 43,328.3 | 34,311.1 | 21,102.8 | 22,630.8 | 27,509.2 |
| Age 20-44 | 65,532.3 | 35,282.3 | 45,240.9 | 36,945.1 | 18,509.0 | 19,966.2 | 26,809.8 |
| Age 45-59 | 72,978.7 | 36,868.0 | 49,905.7 | 38,348.2 | 23,624.7 | 25,614.0 | 28,642.1 |
| Age 60+ | 62,728.9 | 34,678.7 | 40,595.6 | 31,995.9 | 21,289.7 | 22,610.6 | 27,260.5 |

**^1^ Geometric mean**

**Supplementary Table 2 - Hospital characteristics of RSV hospitalizations, 2010 – 2012, compared to Pneumonia virus unspecified**

| Hospital Characteristic | RSV  n (% of total hospitalizations) | Pneumonia virus unspecified  n (% of total hospitalizations) |
| --- | --- | --- |
| Total Hospitalizations | 12,206 | 25,175 |
| Region |  |  |
| NE | 2,409 (24.5%) | 3,386 (16.8%) |
| Midwest | 2,469 (25.2%) | 4,628 (23.0%) |
| South | 2,825 (28.8%) | 7,212 (35.8%) |
| West | 2,110 (21.5%) | 4,902 (24.4%) |
| Location |  |  |
| Rural | 462 (4.8%) | 3,500 (17.5%) |
| Urban nonteaching | 2,012 (20.9%) | 8,081 (40.5%) |
| Urban teaching | 7,131 (74.2%) | 8,379 (42.0%) |
| Bedsize |  |  |
| Small | 1,079 (11.2%) | 2,931 (14.7%) |
| Medium | 1,914 (19.9%) | 4,683 (23.5%) |
| Large | 6,611 (68.8%) | 12,346 (61.9%) |
